# Encapsulation of Cherry Laurel (*Prunus laurocerasus*) Extracts via Spray and Freeze Drying: Effects on Bioactive Compounds, Volatile Profile, and Physicochemical Properties of the Powder

**DOI:** 10.1002/fsn3.71775

**Published:** 2026-04-14

**Authors:** Ahmet Alp Karamanoglu, Zekai Tarakçi, Mehmet Akif Karagol

**Affiliations:** ^1^ Graduate School of Natural and Applied Sciences Ordu University Ordu Turkey; ^2^ Faculty of Agriculture, Department of Food Engineering Ordu University Ordu Turkey

**Keywords:** gum arabic, maltodextrin, PCA, powder properties, SEM, volatile profiling

## Abstract

This study evaluated the encapsulation of cherry laurel ( *Prunus laurocerasus* ) fruit extracts by spray drying and freeze‐drying using gum arabic, maltodextrin, and their mixture. Powder yields ranged from 63.57% (MD165) to 98.33% (MD‐L), with freeze‐dried samples showing higher recovery. Encapsulation efficiencies varied between 23.89% and 83.82%, highest in gum arabic‐coated powders. Moisture contents ranged from 2.85% to 7.55%, with water activity below 0.32 for all samples. The highest total phenolic content (469.14 mg GAE/100 g) and antioxidant capacity (514.39 mg TE/100 g) were observed in freeze‐dried samples. Thirty‐three volatile compounds were identified by GC–MS; alcohols and hydrocarbons were found at higher levels in freeze‐dried samples, whereas ketones were primarily formed in spray‐dried powders. PCA and heatmap analyses confirmed that drying method and coating material significantly affected volatile profiles. PCA score plots clearly separated freeze‐dried and spray‐dried groups, with PC1 and PC2 explaining over 60% of total variance. Heatmap clustering showed gum arabic improved retention of acetic acid and esters, while maltodextrin preserved aldehydes like benzaldehyde. SEM images revealed smooth, spherical particles in spray‐dried samples and porous, fractured structures in freeze‐dried powders. Overall, freeze‐drying combined with gum arabic achieved the best retention of bioactive and volatile compounds, while spray drying with maltodextrin produced powders with good morphology and flowability. These results highlight how encapsulation method and wall material selection optimize the functional quality of cherry laurel powders for use in value‐added functional food products.

## Graphical abstract

Spray drying and freeze drying with different wall materials affected the physicochemical properties, bioactive compounds, volatile profile, and microstructure of encapsulated cherry laurel powders.

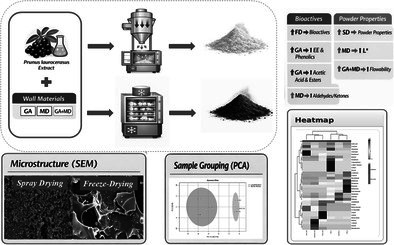

## 1 Introduction

Cherry Laurel ( *Prunus laurocerasus* ) is a fruit species native to the eastern Black Sea region of Turkey, characterized by its rich phenolic content, strong antioxidant capacity, and distinctive aromatic profile. Traditionally consumed fresh or processed into jam, molasses, and pickles, cherry laurel has seen limited utilization in large‐scale industrial applications, suggesting that its potential as a value‐added product remains underutilized (Ayaz et al. 1998; Alasalvar et al. 2005). The literature indicates that cherry laurel fruit is a rich source of vitamin C, total phenolics, anthocyanins, and flavonoids, which provide potent antioxidant, anti‐inflammatory, and anticancer properties (Kolayli et al. 2003; Sahan et al. 2012; Liyana‐Pathirana et al. 2006). Preserving these valuable compounds during processing requires the application of appropriate technological approaches. Freeze‐drying and spray‐drying are promising techniques for minimizing the degradation of bioactive compounds and enhancing the quality of the final product (Ratti 2001; Gharsallaoui et al. 2007). Furthermore, encapsulation offers an effective strategy for improving the stability of bioactive compounds during processing and controlling their release in the final product (Troise and Fogliano 2013). Microencapsulation helps protect the physical, chemical, and biological integrity of active ingredients, minimizing adverse effects during handling and processing (Madene et al. 2006). The use of natural coating materials such as maltodextrin and arabic gum contributes to both product quality and controlled‐release properties (Desai and Jin Park 2005; Gharsallaoui et al. 2007).

The aim of extending shelf life, overcoming seasonal limitations, and ensuring year‐round availability has driven significant advancements in preservation technologies within the food industry. In addition to traditional methods, modern technologies such as drying, irradiation, and modified atmosphere packaging have significantly contributed to improving microbial and chemical stability (Çelik et al. 2020). Maintaining the physical and chemical properties of fresh products during extended storage has become an ongoing expectation among both producers and consumers. This demand has prioritized the development of cost‐effective, efficient, and sustainable preservation techniques within food technology research. Fruits, due to their content of phenolic compounds, carotenoids, vitamins, and other phytochemicals, play an important functional role in human health (Liu 2013). However, their high moisture content makes them particularly vulnerable to microbial spoilage, which limits shelf life and reduces market value (Kian‐Pour 2020). Therefore, preserving the nutritional and bioactive attributes of fruits over extended periods has become a key research objective in food science research.

Drying processes remove free water from products, thereby inhibiting microbial growth and enzymatic activity and ultimately extending shelf life (Ertekin and Yaldiz 2004). Techniques such as sun drying, hot‐air drying, vacuum drying, microwave drying, and freeze‐drying have been developed and widely adopted on an industrial scale (Gao et al. 2012; Türkmen et al. 2020). Among these methods, freeze‐drying is particularly valued for its ability to preserve the color, aroma, and bioactive compounds of products, making it ideal for producing high‐value functional foods (Hammami and René 1997). Previous studies on cherry laurel have also shown that freeze‐drying is more effective than conventional drying methods in preserving phenolic compounds and antioxidant activity (Çelik et al. 2020). In recent years, consumer demand has shifted from products that are merely physically stable to those that also retain their functional and bioactive properties. As a result, preserving fruit‐derived phenolics and antioxidants during processing has become a central focus in functional food development. The integration of advanced drying techniques with encapsulation technologies has proven effective in stabilizing bioactive compounds and maintaining their functional roles within food matrices (Gharsallaoui et al. 2007; Madene et al. 2006). Spray drying was selected in this study due to its rapid operation, economic feasibility, and wide applicability in the production of stable encapsulated powder products (Caliskan and Dirim 2013). The primary objective of this study was to convert *Prunus laurocerasus* (cherry laurel) fruit extract into stable powders via spray drying and freeze‐drying using different wall materials, and to comprehensively characterize the resulting powders. The underlying research problem is that drying technology and formulation conditions may cause losses and compositional shifts in both phenolic bioactives and aroma‐related volatile compounds, which can limit the application of fruit extracts as functional ingredients. We hypothesized that freeze‐drying would better preserve bioactive compounds and volatile constituents, whereas spray drying would provide powders with favorable technological properties, and that wall material selection (maltodextrin and gum arabic) would significantly modulate these outcomes. Therefore, the effects of drying method and wall material on physicochemical properties, total phenolic content, antioxidant activity, and volatile fingerprints were investigated. In addition, SEM was used to examine powder microstructure, while PCA and heatmap visualization were applied to interpret multivariate patterns and group‐level differences in the volatile profiles. Overall, this work contributes new knowledge by providing an integrated evaluation of drying technology–wall material interactions specifically for cherry laurel extract powders, with an emphasis on volatile preservation alongside bioactive retention and powder functionality.

## 2 Materials and Methods

### 2.1 Materials

Cherry laurel fruits ( *Prunus laurocerasus* ) were harvested at consumption maturity in August from a mixed‐species garden in Karapınar village, Ordu. After removing stems and leaves, the fruits were washed, drained, divided into 500‐g portions, sealed in freezer bags, and stored at −18°C. Maltodextrin (DE5, Sigma‐Aldrich) and arabic gum (Vankim Kimya) were used as encapsulation agents.

### 2.2 Chemicals and Reagents

Folin–Ciocalteu reagent and sodium carbonate were obtained from Merck (Darmstadt, Germany). DPPH (2,2‐diphenyl‐1‐picrylhydrazyl), gallic acid, and Trolox were purchased from Sigma‐Aldrich (St. Louis, MO, USA). Methanol was obtained from Merck (Darmstadt, Germany). For retention index (RI) calculations in GC–MS analysis, a Supelco C7–C40 *n*‐alkane standard mixture was used (Supelco, Bellefonte, PA, USA). Volatile compounds were extracted using an SPME fiber assembly (Supelco 57344‐U; 75 μm CAR/PDMS; Supelco, Bellefonte, PA, USA). For SEM analysis, samples were sputter‐coated with gold prior to imaging. All reagents were of analytical grade unless otherwise stated.

### 2.3 Preparation of Fruit Extract

The fruits were heated to 80°C in distilled water (2:1, water to fruit ratio) without prior crushing. Once the target temperature was reached, the fruits were mashed and kept at 80°C for 20 min. The extraction temperature of 80°C was chosen to improve tissue softening and extract recovery during pulp preparation. Nevertheless, this treatment may have contributed to some loss of heat‐sensitive bioactive compounds. The resulting extract was filtered through a metal sieve, cooled, filtered again with muslin cloth, and stored at −18°C. Arabic gum, maltodextrin, and their 1:1 mixture (adjusted to the same °Brix as the extract) were added at a 1:1 ratio to form emulsions, which were homogenized for 10 s using a probe‐type ultrasound homogenizer (Hielscher UP100h).

### 2.4 Encapsulation by Spray Drying and Freeze‐Drying Methods

**FIGURE 1:**
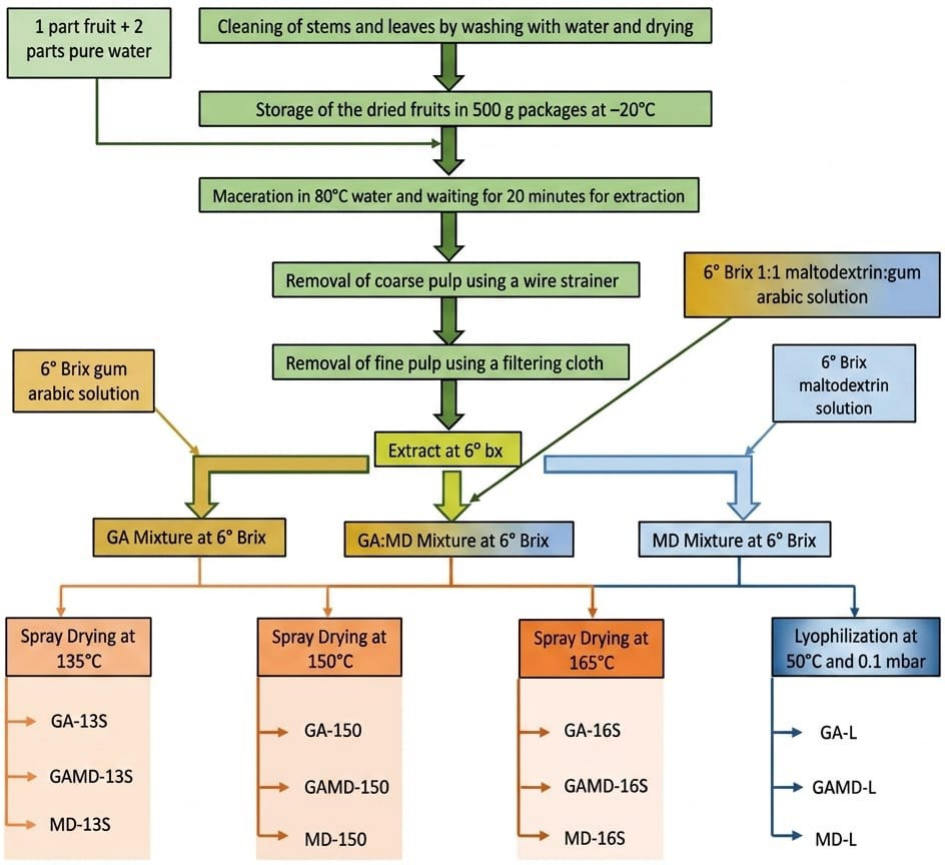
Flow chart for encapsulated powder production.

The spray‐drying and freeze‐drying conditions were selected based on the literature and a partially modified protocol of Balcı‐Torun (2019), considering the operational constraints of the equipment and practical quality criteria such as target moisture/water activity, powder yield (recovery), minimization of stickiness/wall deposition, and maintaining a stable outlet temperature window. Additionally, a small set of preliminary trials was conducted to verify that the selected inlet temperature range provided free‐flowing powders without severe wall deposition. The flow chart illustrating the production process of encapsulated cherry laurel powder is shown in Figure 1. Spray drying was performed using a laboratory‐scale spray dryer (MSK‐USP6000, MTI Corp., USA). According to the manufacturer, the system is equipped with a compressed gas‐assist dual‐channel spray nozzle with an anti‐clogging function, a nozzle diameter of 0.7 mm, a peristaltic pump with an adjustable feeding‐rate range of 30–2200 mL/h, and a drying air flow capacity of 35 m^3^/h. The wall material solutions (gum arabic, maltodextrin, and their 1:1 combination) were adjusted to the same soluble solids content as the cherry laurel extract (6°Brix) before drying. Encapsulation by spray drying was carried out at inlet temperatures of 135°C, 150°C, and 165°C. The fan/aspiration setting was maintained at 100% throughout the drying process, and the cleaning pin interval was set to 1 s. The feed pump setting was 20% at 135°C, 30% at 150°C, and 45% at 165°C. The outlet temperature observed during spray drying was approximately in the range of 75°C–80°C, depending on the inlet temperature and feed conditions. After the addition of coating agents, the emulsions were prepared and subsequently frozen at −20°C for 24 h. The frozen samples were then dried for 6 days (144 h) at −50°C under a pressure of 0.1 mbar using a laboratory‐type lyophilizer (Labconco, USA). A total of 12 encapsulated powder formulations were produced in this study. For spray drying, three wall material formulations (gum arabic, maltodextrin, and their 1:1 combination) were processed at three inlet temperatures (135°C, 150°C, and 165°C), resulting in 9 spray‐dried treatments. In addition, the same three wall material formulations were also subjected to freeze‐drying, resulting in 3 freeze‐dried treatments. Thus, 12 powder samples were obtained in total. Analytical measurements for each sample were carried out in three parallel replicates (*n* = 3).

### 2.5 Analysis of the Physical and Chemical Attributes of Fresh Fruit Pulp and Extract

The dry matter content of the cherry laurel pulp was determined according to the AOAC (2020) method. The soluble solid content was measured using a digital refractometer (Hanna Instruments 96801), and the pH values were measured with a laboratory‐type pH meter (FE20—Five Easy, Mettler‐Toledo, Ohio, USA). Titratable acidity was determined by titrating 9.2 mL of the extract obtained from cherry laurel pulp to pH 8.1 with standardized NaOH solution using a standard burette‐based titration setup. The volume of NaOH consumed during titration was recorded, and titratable acidity was expressed as grams of malic acid per 100 g of sample (Cemeroğlu 2010). The color properties of the samples were determined using a CR‐400 chromameter (Konica Minolta, Japan), and the results were reported in terms of L*, a*, and b* values. The total phenolic content in cherry laurel fruit was determined according to the method described by Škerget et al. (2005), while the DPPH radical scavenging activity was determined according to the method described by Fernández‐León et al. (2013). All analyses were performed in triplicate (*n* = 3).

### 2.6 Determination of Physicochemical and Bioactive Properties of Encapsulated Fruit Powders

Powder yield refers to the mass of powder obtained (recovered) after the drying process. It was calculated according to the method described by Bazaria and Kumar (2017). Bulk density, tapped density, and Carr Index (CI) values were determined based on the procedure outlined by Rege et al. (2003). For this purpose, 2 g of the prepared cherry laurel powder was weighed into a 10 mL graduated cylinder. The cylinder was tapped once on a stable surface, and the volume was recorded to determine the bulk density (ρb, kg/m^3^). Subsequently, the cylinder was tapped 40 times, and the new volume was recorded to calculate the tapped density (ρt, kg/m^3^). Each analysis was carried out in three analytical replicates (*n* = 3). These values were then used to calculate the Carr Index (CI) using the following formula (1):

(1) $$\mathrm{CI}=\left(\left(\mathrm{\rho t}-\mathrm{\rho b}\right)/\mathrm{\rho t}\right)\times 100$$

ρt = tapped density (kg/m^3^); ρb = bulk density (kg/m^3^).

### 2.7 Determination of Solubility, Moisture Content, Water Activity, and Color Properties of Encapsulated Fruit Powders

The solubility of the powders was determined according to the method described by Nadeem et al. (2011). For this purpose, 0.50 ± 0.001 g of sample was weighed, and 50 mL of water was added. The mixture was stirred using a magnetic stirrer (Jeio Tech MS‐32M) at 600 rpm for 5 min. The resulting solution was transferred into centrifuge tubes and centrifuged at 3000 g for 5 min (Herolab Unigen 111 MR, Germany). After centrifugation, 20 mL of the supernatant was transferred to pre‐weighed petri dishes and dried at 70°C until reaching a constant weight. Solubility (%) was calculated based on the weight difference.

The moisture content of the encapsulated powders was determined using a moisture analyzer (RADWAG MAC 50 LCD/Halogen) at 105°C. Water activity was measured with a water activity meter (Aqua Lab Dew Point Water Activity Meter 4TE). The color properties of the powders were determined using a CR‐400 Chromameter (Konica Minolta, Japan), and the results were reported as L*, a*, and b* values. Each analysis was carried out in three analytical replicates (*n* = 3).

### 2.8 Characterization of Phenolic Content (Total and Surface) and Encapsulation Efficiency

For the dissolution of encapsulated powders, 1 g of sample was mixed with 20 mL of a methanol–water solution (containing 0.1% HCl) at a ratio of 80:20 (*v*/*v*) and vortexed for 3 min. After extraction in a water bath at 60°C for 20 min, the samples were centrifuged at 4000 rpm for 5 min. Then, 0.5 mL of the supernatant was taken into glass tubes, and the total phenolic content was determined according to the method described by Škerget et al. (2005).

For the determination of surface phenolic content, 1 g of sample was dissolved in 10 mL of methanol and vortexed at 2000 rpm for 3 s, targeting only the dissolution of surface phenolics. The samples were then centrifuged at 4000 rpm for 5 min, and 0.5 mL of the supernatant was used for phenolic content determination in the same procedure as the total phenolic analysis (Coşgun et al. 2024).

Encapsulation efficiency (EE%) of the powders was calculated based on the difference between the total and surface phenolic content using the following Equation (2) (Özdemir 2019):

(2) $$\text{Encapsulation Efficiency}\;\left(\%\right)=\frac{\left(\mathrm{TF}-\mathrm{YF}\right)\times 100}{\mathrm{TF}}$$

TF = total phenolic content; YF = surface phenolic content.

All total phenolic content, surface phenolic content, and encapsulation efficiency determinations were carried out in triplicate (*n* = 3).

### 2.9 Determination of Antioxidant Activity by the DPPH Assay

Antioxidant activity of the encapsulated powder samples was determined using the DPPH radical scavenging assay. For extract preparation, 0.5 g of encapsulated powder samples was dissolved in 24.5 mL of a methanol: water solution (containing 0.1% HCl) at a ratio of 80:20 (*v*/*v*). The DPPH solution was prepared by dissolving 59.2 mg of DPPH in 25 mL of methanol and adjusting the absorbance to fall within the range of 0.680–0.720. For the DPPH assay, 50 μL of the extract was mixed with 950 μL of 6 × 10^−5^ M DPPH solution, vortexed, and incubated in the dark at room temperature for 30 min. Absorbance was measured at 516 nm (Shimadzu UV–Vis 160A, Japan), using methanol as a blank. Results were expressed as mg Trolox equivalents per 100 g of sample (Fernández‐León et al. 2013). Each analysis was carried out in triplicate (*n* = 3).

### 2.10 Determination of Total Monomeric Anthocyanin Content

The total monomeric anthocyanin content of the encapsulated powders was quantified using the pH differential method, which exploits the structural transformation of anthocyanins at different pH levels. In this method, anthocyanins predominantly exist in the colored oxonium form at pH 1.0 and in the colorless hemiketal form at pH 4.5. Absorbance measurements are taken at both pH values, and the anthocyanin concentration is calculated based on the difference in absorbance between the two buffer conditions. To perform these measurements, buffer solutions (potassium chloride and Sodium acetate) were prepared (Giusti and Wrolstad 2001).

Preliminary trials determined that the maximum absorbance for cherry laurel anthocyanins occurred at 514 nm, and measurements were also taken at 700 nm to eliminate the effect of turbidity. Absorbance readings were taken against distilled water as the blank. The total monomeric anthocyanin content was calculated using the following Equation (3) and expressed as mg/100 g after multiplying by 10^−1^. The analysis was performed in triplicate (*n* = 3).

(3) $$\text{Total Monomeric Anthocyanin}\;\left(\mathrm{mg}/L\right)=\frac{A\times \mathrm{MW}\times \mathrm{SF}\times 10^{3}\#}{ɛ\times l}$$

*A* = absorbance difference = (*A*_max_ − A_700_) pH 1.0 − (A_max_ − A_700_) pH 4.5.

MW = molecular weight (440.2 for cyanidin‐3‐glucoside).

DF = dilution factor.

*ε* = molar absorptivity (26,900 for cyanidin‐3‐glucoside).

*l* = path length of the cuvette (cm).

### 2.11 Determination of Volatile Organic Compounds

The volatile compounds of the cherry laurel powder samples were determined using Gas Chromatography–Mass Spectrometry (GC–MS, GCMS‐QP2010 Ultra, Shimadzu, Milan, Italy). The solid‐phase microextraction (SPME) method, as described and modified from Triaux et al. (2019), was applied. The SPME auto‐sampler (AOC‐6000, Shimadzu, Tokyo, Japan) was connected to the GC–MS injector. Three grams of each sample were weighed into 20‐mL glass headspace vials sealed with PTFE/silicone septa. The samples were first heated at 60°C for 40 min, and then the fiber was exposed to the headspace of the vial for 20 min to allow absorption of volatile compounds onto the fiber. The SPME injection parameters were set as follows: needle penetration 12 mm, fiber penetration 10 mm, extraction time 10 min, injection penetration 26 mm, desorption time 5 min, and a total runtime of 52 min.

Chromatographic separation was performed using helium as the carrier gas on an Rxi‐5MS capillary column (30 m × 0.25 mm; film thickness 0.25 μm). The oven temperature program was as follows: initial hold at 40°C for 2 min, ramp to 200°C at 4°C/min over 40 min, hold at 200°C for 2 min, ramp to 230°C at 10°C/min over 3 min, and a final hold at 230°C for 5 min. The carrier gas pressure was maintained at 49.7 kPa, with a linear velocity of 36.1 cm/s and a flow rate of 1.0 mL/min. The interface temperature was set at 250°C and the ion source temperature at 200°C. Volatile compounds were identified using the NIST and Wiley mass spectral libraries, and the results were expressed as “peak area × 10^−6^.” Retention indices (RI) were calculated using the Supelco C7–C40 *n*‐alkane standard mixture and were used to support compound identification by comparison with library/reference RI data. The effects of different drying methods and the use of encapsulation materials on the identified volatile compounds were determined using heatmap and Principal Component Analysis (PCA), performed with the online software MetaboAnalyst. Volatile compound analysis was carried out in duplicate (*n* = 2).

### 2.12 Scanning Electron Microscopy Imaging

Scanning Electron Microscopy (SEM) was used to observe the effectiveness of the coating materials and the impact of the drying method on the structure of the cherry laurel powder samples. Images were captured at magnifications of 500× and 8000×. For better image quality, powder samples were fixed onto aluminum stubs with double‐sided adhesive tape and sputter‐coated with gold prior to imaging in order to enhance electrical conductivity and obtain clear surface micrographs. A Hitachi SEM device (SU1510 model) was used for image acquisition.

### 2.13 Statistical Analysis

Powders were produced by spray drying and freeze‐drying using different wall material formulations (maltodextrin and gum arabic combinations). In total, 12 different powder samples/formulations were obtained and used for physicochemical and bioactive/antioxidant analyses, while volatile profiling was performed on 6 selected samples. Physicochemical and bioactive analyses were carried out in three parallel determinations (*n* = 3), whereas volatile compound analysis was performed in duplicate (*n* = 2). The resulting data were statistically analyzed using SPSS statistical software, version 25 (IBM Corp., Armonk, NY, USA). Results are expressed as mean values ± standard deviation, with a significance level set at *p* < 0.05. Prior to parametric testing, normality and homogeneity of variance were assessed using the Shapiro–Wilk and Levene's tests, respectively, and no violations were detected. Therefore, each measured parameter was subjected to one‐way analysis of variance (one‐way ANOVA), and significant differences among means were determined using Tukey's multiple comparison test and expressed with different letters.

## 3 Result and Discussion

### 3.1 Cherry Laurel Pulp Analysis Results

The physicochemical and bioactive properties of the cherry laurel pulp used in this study are presented in Table 1. The values presented in Table 1 refer to the cherry laurel pulp obtained by homogenizing the fruit prior to the 80°C heat treatment step. The dry matter content (21.45%) and soluble solids content (18.87°Brix) were generally in agreement with values previously reported for cherry laurel fruits (Kaya and Aydın 2007; Üstün and Tosun 2003; İslam and Deligöz 2012; Islam and Vardal 2006; Akbulut et al. 2007). The pH (4.65) and titratable acidity (0.251% malic acid) values also confirmed the acidic character of the fruit, although some variation from the literature may be expected depending on genotype, maturity stage, and processing conditions. In terms of bioactive properties, the total phenolic content and antioxidant activity determined in the present study differed from some previously reported values, which may be attributed to differences in extraction conditions, sample preparation, and expression of results. For example, Temiz and Tarakçı (2017) reported cherry laurel pulp values of 16% dry matter, pH 3.9, titratable acidity 0.63% malic acid, and total phenolic content of 1052.73 mg/kg GAE, while Karabegovic et al. (2013) reported 11.07 mg GAE/100 g for cherry laurel fruit. The color values obtained in this study (L*, a*, and b*) also reflect the dark reddish‐purple nature of cherry laurel and are useful as baseline characteristics for interpreting the properties of the encapsulated powders.

**TABLE 1:** Physicochemical values of cherry laurel fruit.

Dry matter (%)	21.45 ± 0.05
Soluble solids content (°Brix)	18.87 ± 0.04
pH	4.65 ± 0.01
Titratable acidity (% malic acid)	0.251 ± 0.003
Total phenolic content (mg GAE/100 g)	13.68 ± 0.08
DPPH radical scavenging activity (mg TE/100 g)	9.57 ± 0.06
L*	22.51 ± 0.28
a*	5.79 ± 0.07
b*	3.73 ± 0.13

*Note:* Values are expressed as mean ± standard deviation.

### 3.2 Physicochemical Analysis Results of Encapsulated Cherry Laurel Powders

The physicochemical analysis results of cherry laurel powders are presented in Table 2. When powder yield was evaluated, GA‐L, MD‐L, and GAMD‐L were among the highest‐yielding samples, and no statistically significant difference was found among them. Overall, freeze‐dried samples showed significantly higher powder yield than spray‐dried samples. From the perspective of drying methods, it was found that the powder yields of the samples produced by lyophilization were higher compared to those produced by spray drying. Overall, freeze‐dried samples exhibited higher powder yield than spray‐dried samples. The effect of inlet temperature on powder yield was not uniform across all spray‐dried formulations; however, in the maltodextrin‐based powders, the sample produced at 165°C showed a lower yield than those produced at 135°C and 150°C. Similarly, in their study on the encapsulation of mountain tea extract, Nadeem et al. (2011) reported that increasing the inlet temperature during drying led to a reduction in powder yield and an increase in moisture content. This phenomenon may be attributed to the increased feed rate required to maintain a stable outlet temperature as the inlet temperature rises, resulting in insufficiently dried particles adhering to the inner walls of the dryer, and in cases where particles do not adhere, their moisture content not being removed as effectively as in lower‐temperature drying processes.

**TABLE 2:** Physicochemical analysis results of cherry laurel powders.

Samples	Powder yield (%)	Encapsulation efficiency (%)	Moisture content (%)	Carr Index (kg/m^3^ × 100)	Bulk density (kg/m^3^)	Tapped density (kg/m^3^)	Solubility (%)	Water activity (*a*_*w*_)
GA135	76.38 ± 1.96^b^	83.82 ± 0.93^f^	3.55 ± 0.07^ab^	23.79 ± 1.71^b^	0.323 ± 0.011^bc^	0.424 ± 0.005^f^	99.50 ± 0.07^c^	0.201 ± 0.04^ab^
GAMD135	78.47 ± 2.94^b^	55.51 ± 1.66^c^	4.75 ± 1.20^abcd^	24.66 ± 0.47^b^	0.279 ± 0.010^a^	0.371 ± 0.015^bcde^	99.22 ± 0.10^c^	0.185 ± 0.03^a^
MD135	80.71 ± 3.03^b^	32.15 ± 2.19^ab^	6.70 ± 1.13^de^	18.29 ± 1.62^ab^	0.286 ± 0.003^a^	0.351 ± 0.002^abc^	98.20 ± 0.77^bc^	0.160 ± 0.00^a^
GA150	77.77 ± 1.96^b^	75.30 ± 2.65^ef^	4.30 ± 0.42^abcd^	18.18 ± 6.21^ab^	0.339 ± 0.013^bc^	0.416 ± 0.014^ef^	97.65 ± 0.56^bc^	0.165 ± 0.02^a^
GAMD150	79.86 ± 0.98^b^	34.48 ± 3.14^ab^	4.65 ± 0.21^abcd^	21.88 ± 3.45^ab^	0.279 ± 0.001^a^	0.357 ± 0.018^abcd^	99.20 ± 0.21^c^	0.192 ± 0.00^a^
MD150	77.14 ± 2.02^b^	23.93 ± 0.97^a^	5.70 ± 0.00^bcde^	18.33 ± 2.35^ab^	0.283 ± 0.005^a^	0.347 ± 0.017^ab^	96.85 ± 0.42^bc^	0.181 ± 0.04^a^
GA165	73.61 ± 3.92^b^	71.48 ± 1.50^de^	4.75 ± 0.91^abcd^	14.03 ± 0.34^a^	0.351 ± 0.008^c^	0.408 ± 0.011^ef^	96.22 ± 0.74^ab^	0.199 ± 0.02^ab^
GAMD165	72.22 ± 3.92^ab^	40.33 ± 2.88^b^	5.10 ± 0.84^abcde^	19.05 ± 0.42^ab^	0.323 ± 0.005^bc^	0.399 ± 0.008^def^	98.50 ± 0.70^bc^	0.213 ± 0.01^ab^
MD165	63.57 ± 3.03^a^	23.89 ± 0.95^a^	7.55 ± 0.49^e^	17.74 ± 2.28^ab^	0.326 ± 0.002^bc^	0.396 ± 0.008^cdef^	97.55 ± 1.97^bc^	0.177 ± 0.01^a^
GA‐L	96.68 ± 0.75^c^	83.42 ± 3.22^f^	6.10 ± 0.14^cde^	16.63 ± 1.42^ab^	0.307 ± 0.012^ab^	0.369 ± 0.008^abcde^	99.55 ± 0.14^c^	0.295 ± 0.02^bc^
MD‐L	98.33 ± 0.39^c^	79.55 ± 5.26^ef^	2.85 ± 0.07^a^	14.08 ± 0.28^a^	0.284 ± 0.005^a^	0.331 ± 0.007^ab^	96.32 ± 0.24^ab^	0.318 ± 0.00^c^
GAMD‐L	98.29 ± 0.44^c^	63.32 ± 5.30^cd^	3.70 ± 0.28^abc^	13.70 ± 0.26^a^	0.279 ± 0.008^a^	0.323 ± 0.011^a^	93.97 ± 0.10^a^	0.319 ± 0.02^c^

*Note:* Values are expressed as mean ± standard deviation. The letters a–f indicate significant differences among the sample groups (*p* < 0.05).

The amount of surface phenolic compounds refers to the phenolic constituents that remain on the surface of microcapsules during the encapsulation process. This term indicates phenolic compounds that are either not completely coated by the encapsulating agent or remain on the outer surface of the coating material, thus not being fully isolated from the external environment. Upon examining the results, it was observed that powders coated with gum arabic exhibited better encapsulation efficiency compared to those coated with maltodextrin or a combination of coating materials. The increase in temperature during the spray‐drying process negatively affected the encapsulation efficiency. Kaya (2021), in a study on encapsulating the antioxidant components of cranberry at different temperatures, reported that a decrease in the ratio of gum arabic to maltodextrin in the encapsulation matrix led to a reduction in encapsulation efficiency. The lowest encapsulation efficiency was observed in the sample subjected to the highest air inlet temperature during spray drying. Akdeniz et al. (2018), in their study on the encapsulation of onion phenolics using maltodextrin and gum arabic as wall materials, found that increasing the ratio of gum arabic led to higher total phenolic content and lower surface phenolic content.

Moisture content is one of the most critical factors affecting product shelf life and storage stability. An increase in moisture content can raise microbiological risks and also influence physical properties such as solubility and bulk density. The moisture content of the encapsulated cherry laurel powders ranged from 2.85% to 7.55%. Similarly, Silva et al. (2013), in a study where they used gum arabic and maltodextrin as encapsulating agents in spray drying, reported moisture contents of the resulting powders between 5.45% and 6.76%.

The bulk density of encapsulated powders is mainly influenced by factors such as moisture content, particle size, chemical composition, and drying‐storage conditions (Bae and Lee 2008). Low bulk density is considered undesirable, as it increases the packaging volume of the product and consequently raises logistical and packaging costs. Additionally, low bulk density implies higher air content in the final product, which increases the risk of oxidation and poses a threat to storage stability (Türker et al. 2018). In this study, the bulk density of the samples ranged from 0.279 to 0.351 kg/m^3^. The effect of sample type on bulk density was found to be statistically significant (*p* < 0.05). Analysis of the results revealed that the drying method had a considerable impact on bulk density, with samples produced by spray drying exhibiting higher values than those obtained through lyophilization. Additionally, powders encapsulated with gum arabic demonstrated higher bulk density compared to those encapsulated with maltodextrin.

Tapped density refers to the bulk density obtained after mechanical compaction of a powder sample through repeated tapping. Evaluation of the tapped density values, the GAMD‐L sample exhibited the lowest value (0.323 kg/m^3^), whereas the GA135 sample demonstrated the highest density (0.424 kg/m^3^). The effect of sample type on tapped density was also found to be statistically significant (*p* < 0.05). Samples produced via spray drying displayed higher tapped density values than those obtained through lyophilization. In terms of encapsulation material, powders produced with gum arabic showed higher tapped density compared to those produced with maltodextrin. This can be attributed to structural differences induced by the encapsulation materials. SEM images confirmed that samples encapsulated with gum arabic exhibited a more wrinkled morphology, while those with maltodextrin showed a smoother and more spherical shape. These morphological differences influence the filling of inter‐particle voids upon tapping, which affects the resulting tapped density. The lower tapped density observed in lyophilized samples is thought to result from the formation of a more fractured structure, as supported by SEM observations.

The flowability of powder products is evaluated using the Carr Index (CI). According to Carr (1965), CI values below 15 indicate excellent flowability, values between 15 and 20 are considered good, 20–35 indicate fair, 35–45 poor, and values above 45 are classified as very poor. When Carr Index values were analyzed, the lowest value was recorded in the GAMD‐L sample (13.70), and the highest in the GAMD135 sample (24.66). In general, lyophilized cherry laurel samples showed lower CI values compared to other samples, and this difference was statistically significant (*p* < 0.05). Based on the results, it can be concluded that the powders encapsulated using freeze‐drying had better flowability than those produced via spray drying. Among the spray‐dried samples, higher processing temperatures resulted in powders with improved flow properties. Furthermore, in the freeze‐drying method, the combination of gum arabic and maltodextrin yielded better flowability results than when these materials were used individually.

In food powders, solubility refers to the ability of the powdered material to dissolve in a given solvent (typically water) to form a homogeneous solution. This property is a key parameter for determining the rehydration capability and overall quality of the final product (Fang et al. 2007). Upon examining the solubility values, the highest was found in the GA‐L sample (99.55%), while the lowest was observed in the GAMD‐L sample (93.97%). These findings suggest that the freeze‐drying method positively influences the solubility of the final product compared to the spray‐drying method. Additionally, in both drying methods, powders encapsulated solely with gum arabic exhibited better solubility properties, while those produced using only maltodextrin had lower water solubility. According to variance analysis, the effect of sample type on solubility was found to be statistically significant (*p* < 0.05).

Regarding water activity (*a*_*w*_), the values of the samples ranged from 0.160 to 0.319. Based on the results of variance analysis, the effect of sample type on water activity was found to be statistically significant (*p* < 0.05). In general, freeze‐dried powders exhibited higher water activity values than those produced by spray drying. Kaya (2021), in her study on the encapsulation of cranberry powders using gum arabic and maltodextrin, reported water activity values for the obtained particles in the range of 0.22–0.27.

Bioactive and color properties of cherry laurel powders are presented in Table 3. Antioxidant activity determined by the DPPH assay differed significantly among the samples (*p* < 0.05), with freeze‐dried powders generally exhibiting higher values than spray‐dried powders. Overall, the freeze‐dried samples exhibited higher antioxidant activity than the spray‐dried samples. This finding agrees with Çelik et al. (2020), who reported that lyophilized cherry laurel showed higher antioxidant activity than vacuum‐ and fan oven‐dried samples. Among the samples, the lowest antioxidant activity was observed in MD150 (259.51 mg TE/100 g), whereas the freeze‐dried samples, particularly GA‐L (483.29 mg TE/100 g) and MD‐L (514.39 mg TE/100 g), were among the samples with the highest antioxidant activity. Upon evaluation of the results, it is evident that the DPPH radical scavenging activity of the lyophilized samples was generally higher than that of the spray‐dried samples. This trend appears to be associated with the retention of phenolic compounds since samples with higher total phenolic content also tended to exhibit higher antioxidant activity. In addition, anthocyanin retention may have contributed to the antioxidant response of the powders, although this relationship was not uniform for all formulations. Therefore, the differences in antioxidant activity should be interpreted as the combined result of drying conditions, wall material composition, and the retention of phenolic and anthocyanin compounds, rather than as the effect of processing temperature alone. Similar observations were reported by Tonon et al. (2010), who showed that antioxidant activity and anthocyanin stability in spray‐dried fruit powders were influenced by both processing conditions and carrier‐related factors.

**TABLE 3:** Bioactive and color properties of cherry laurel powders.

Samples	DPPH radical scavenging activity (mg TE/100 g)	Total monomeric anthocyanins (mg/100 g)	Total phenolic content (mg GAE/100 g)	L*	a*	b*
GA135	367.43 ± 2.58^cd^	59.41 ± 8.61^c^	405.60 ± 3.08^cd^	71.50 ± 1.45^d^	16.51 ± 0.73^a^	0.38 ± 0.01^b^
GAMD135	310.73 ± 27.59^abc^	37.90 ± 6.16^b^	312.46 ± 9.44^abc^	72.32 ± 1.22^d^	17.46 ± 0.82^ab^	1.27 ± 0.26^c^
MD135	285.12 ± 1.72^ab^	52.26 ± 2.44^bc^	293.91 ± 69.23^a^	69.48 ± 0.43^d^	19.56 ± 0.21^bcd^	−0.39 ± 0.05^a^
GA150	323.53 ± 9.48^bc^	54.07 ± 0.63^bc^	238.69 ± 6.94^a^	71.24 ± 0.94^d^	17.79 ± 0.95^ab^	0.32 ± 0.09^b^
GAMD150	274.75 ± 2.58^ab^	48.46 ± 1.43^bc^	292.82 ± 33.36^a^	70.96 ± 0.35^d^	18.10 ± 0.32^ab^	1.36 ± 0.22^c^
MD150	259.51 ± 8.62^a^	60.31 ± 5.53^c^	232.55 ± 27.96^a^	68.95 ± 2.19^cd^	19.53 ± 1.17^bcd^	0.14 ± 0.01^b^
GA165	349.75 ± 12.07^cd^	47.22 ± 7.65^bc^	220.14 ± 13.11^a^	70.25 ± 0.29^d^	18.45 ± 0.41^abc^	0.36 ± 0.06^b^
GAMD165	402.80 ± 18.10^de^	46.36 ± 2.75^bc^	257.78 ± 1.15^a^	68.43 ± 0.26^cd^	19.05 ± 0.20^bcd^	1.62 ± 0.24^c^
MD165	323.53 ± 15.34^bc^	62.49 ± 3.61^c^	297.60 ± 1.92^ab^	65.24 ± 1.81^c^	21.47 ± 0.87^d^	0.12 ± 0.01^ab^
GA‐L	483.29 ± 25.00^f^	8.15 ± 0.05^a^	460.96 ± 15.04^d^	48.57 ± 0.30^a^	18.15 ± 0.16^ab^	2.73 ± 0.03^d^
MD‐L	514.39 ± 3.44^f^	13.16 ± 0.95^a^	400.14 ± 32.39^bcd^	53.50 ± 0.23^b^	20.92 ± 0.01^cd^	1.60 ± 0.021^c^
GAMD‐L	461.95 ± 22.42^ef^	15.56 ± 2.97^a^	469.14 ± 7.32^d^	45.87 ± 0.49^a^	17.34 ± 0.11^ab^	2.38 ± 0.11^d^

*Note:* Values are expressed as mean ± standard deviation. The letters a–f indicate significant differences among the sample groups (*p* < 0.05).

Regarding the anthocyanin content of the powder samples, the lowest value was observed in the GA‐L sample (8.15 mg/100 g), while the highest value was recorded in the MD165 sample (62.49 mg/100 g). The effect of sample type on anthocyanin content was found to be statistically significant (*p* < 0.05). In a study by Alasalvar et al. (2005) involving two different cherry laurel cultivars, the total anthocyanin content was reported between 123 and 174 mg/100 g. Similarly, Özbey (2009) determined the anthocyanin content as 52.05 mg/100 g for red‐colored cherry laurel and 105.83 mg/100 g for purple‐colored cherry laurel. The relationship between total anthocyanins and color parameters is consistent with findings in other studies. The lower total anthocyanin content in the lyophilized samples is thought to result from degradation caused by oxidation due to insufficient encapsulation efficiency.

The total phenolic content of the samples ranged from 220.14 to 469.14 mg GAE/100 g. Among the samples, GAMD‐L exhibited the highest phenolic concentration, while GA165 showed the lowest. Statistical analysis revealed that the sample type had a significant effect on total phenolic content (*p* < 0.05). Comparable findings have been reported in the literature. For instance, Çelik et al. (2020) evaluated the impact of different drying techniques on the phenolic profile of cherry laurel and determined a total phenolic content of 335.99 mg GAE/100 g for lyophilized samples. Similarly, in an earlier study assessing the physicochemical characteristics of various cherry laurel genotypes, Çelik et al. (2011) reported total phenolic values ranging from 364 to 503 mg GAE/100 g, which corroborate the findings of the present work. Furthermore, Türkmen et al. (2020) found a total phenolic content of 311.88 mg GAE/100 g when cherry laurel fruits were subjected to hot air drying at 50°C, further supporting the variation in phenolic retention depending on processing conditions.

The L* values of the samples ranged from 45.87 to 72.32. According to the results of variance analysis, the effect of sample type on L* values was statistically significant (*p* < 0.05). It was observed that the L* values of lyophilized powders were lower than those produced by spray drying. This is likely due to the degradation of anthocyanin pigments caused by the high temperatures applied during spray drying, which results in higher L* values for encapsulated powders produced using this method (Saikia et al. 2015). Furthermore, among the spray‐dried powders, samples encapsulated with maltodextrin exhibited higher L* values compared to others, likely due to the naturally white color of maltodextrin. The a* values of the samples ranged from 16.51 to 21.47, and according to variance analysis, the effect of sample type on a* values was also statistically significant (*p* < 0.05). When comparing a* values, the effect of the encapsulating material was found to be more prominent than that of the drying method. In general, samples encapsulated with gum arabic had lower a* values compared to those encapsulated with maltodextrin. The b* values ranged from −0.13 to 2.73. According to variance analysis, the effect of sample type on b* values was statistically significant (*p* < 0.05). The b* values of lyophilized powders were found to be higher than those of powders produced by spray drying. In the study conducted by Saikia et al. (2015), where phenolic compounds extracted from *Averrhoa carambola* (starfruit) were encapsulated using both spray drying and freeze‐drying methods, it was observed that the L* and a* values were higher in the spray‐dried powders, whereas the b* values were comparatively lower than those obtained from freeze‐dried powders. Similarly, in a study focusing on the freeze‐drying of cherry laurel ( *Prunus laurocerasus* ) fruit, Dirim and Talih (2018) reported that increasing the proportion of gum arabic in the encapsulation matrix resulted in elevated b* values. This outcome was attributed to the intrinsic yellow hue of gum arabic, which influenced the overall color characteristics of the final powder.

### 3.3 Scanning Electron Microscope (SEM) Images of Cherry Laurel Powder Samples

**FIGURE 2:**
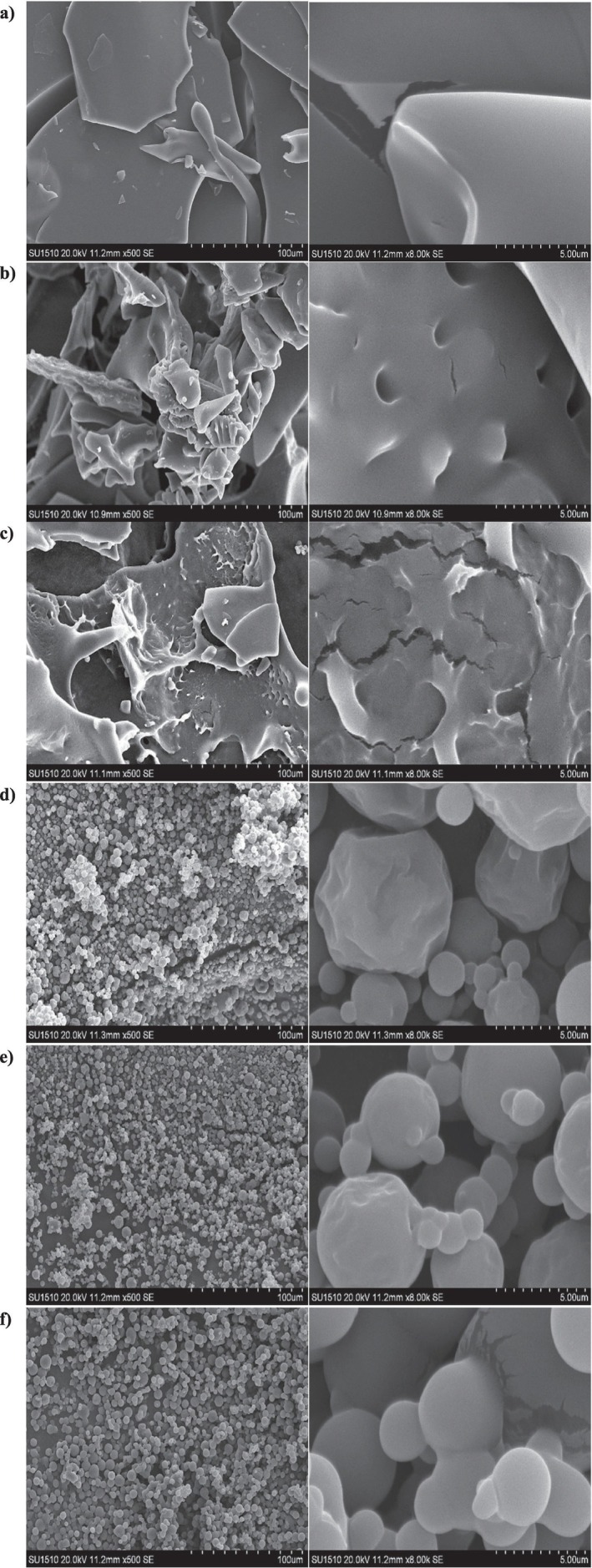
Scanning electron microscope images of the samples (at 500× and 8000× magnifications, respectively): GA‐L (a), GAMD‐L (b), MD‐L (c), GA‐135 (d), GAMD‐135 (e), MD‐135 (f).

In microencapsulation, the desired structures are those with smooth surfaces, intact spherical shapes, and minimal cracks, depressions, or protrusions (Barros and Stringheta 2006). The scanning electron microscope (SEM) images of the powder samples are presented in Figure 2. When comparing the three different powders produced via spray drying and powders produced via freeze‐drying, it was observed that the spray‐dried powders exhibited spherical morphologies, whereas the freeze‐dried powders appeared as irregular, fractured glass‐like forms. Although surface depressions were noted in the spray‐dried powders encapsulated with gum arabic, no cracks or fractures were present. Such structural integrity is desirable, as it results in low gas permeability between the encapsulated core material and the external environment, contributing to the stability of the encapsulated product and enabling controlled release.

Studies have reported that surface depressions in powder capsules can occur due to the rapid shrinkage of droplets caused by sudden heat exposure during the initial phase of drying, and due to the rapid removal of water (Cano‐Higuita et al. 2015; Khazaei et al. 2014). In the spray drying process, when encapsulation was carried out using only maltodextrin, smoother surfaces were observed. In contrast, encapsulation using only gum arabic resulted in more wrinkled capsule surfaces. Similar findings were reported by Kanakdande et al. (2007), who encapsulated cumin oleoresin extract with maltodextrin and gum arabic. This difference is thought to arise from the larger molecular size of gum arabic compared to maltodextrin.

The encapsulated powders obtained through freeze‐drying exhibited irregular, fractured glass‐like structures with a porous surface. During the initial stage of the freeze‐drying process, the extracts are frozen in combination with different wall materials. During the drying phase, the sublimation of water in the frozen matrix—without passing through the liquid phase—leads to the formation of pores and cracks on the microcapsules. The cracks observed on the outer walls of the microcapsules are considered undesirable in encapsulation, as they can negatively affect the stability of the encapsulated material due to increased gas permeability (Santana et al. 2016).

In a study on the encapsulation of turmeric plant extract, it was reported that powders produced by spray drying exhibited spherical and smooth shapes with no visible cracks or pores, whereas microcapsules obtained through freeze‐drying appeared as sharp, fractured glass‐like structures and were reduced to irregular particles after grinding (Cano‐Higuita et al. 2015). Similarly, Chen et al. (2013) observed comparable morphological differences between microcapsules produced by spray drying and freeze‐drying techniques.

Based on these findings, it is evident that different drying methods result in distinct surface morphologies of microcapsules. The presence of smooth, pore‐free, crack‐free, and compact surfaces on microcapsules is considered an indicator of successful encapsulation.

### 3.4 Volatile Compounds Identified in Cherry Laurel Powders

Most fruits produce a significant amount of volatile compounds as they ripen. Although many of these compounds are present in very low concentrations—often below the detection limits of most instruments—they can still be perceived by the human sense of smell (Goff and Klee 2006). Alcohols and aldehydes are key contributors to the aroma and flavor of fruits and also act as precursors in ester synthesis. Therefore, their composition reflects the ester profile of the fruit (Song and Forney 2008). The analysis of volatile compounds from both intact and disrupted fruit tissues can affect aroma profiles and sensory interpretation. Volatiles from intact fruits reflect ripening signals perceived by smell, while those from disrupted tissues better represent flavor during consumption. Since some volatiles formed after tissue breakdown may be transient, it is essential to develop and standardize sampling methods that accurately reflect the compounds released during mastication (Tikunov et al. 2005).

**FIGURE 3:**
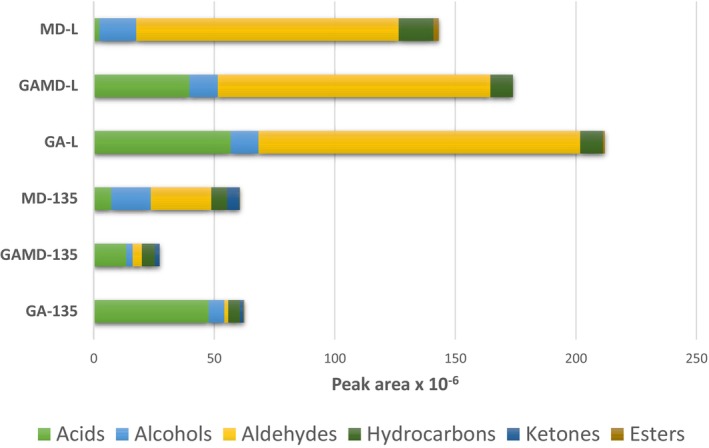
Volatile compound distribution of cherry laurel powder samples.

Volatile Compounds Identified in Encapsulated Cherry Laurel Powders are presented in Table 4. In the present study, volatile compounds in cherry laurel powder samples were identified using solid‐phase microextraction (SPME) coupled with GC–MS. A total of 33 volatile compounds were detected, including acids (1), alcohols (8), aldehydes (8), esters (2), hydrocarbons (12), and ketones (2). The distribution of the volatile compounds identified in the powder samples is shown in Figure 3. As illustrated, powders encapsulated with gum arabic exhibited higher levels of acidic volatiles compared to other samples. Additionally, aldehyde compounds were found in greater quantities in the samples dried by lyophilization. Ketone compounds, on the other hand, were detected only in the samples produced using spray drying. Analysis revealed the presence of eight volatile alcohol compounds: 2‐butanol, 1‐butanol, 2‐hexanol, 2,3‐butanediol, benzyl alcohol, 2‐ethyl‐1‐hexanol, 2‐phenylethanol, and 1‐dodecanol. Considering total concentrations, the highest alcohol content was observed in the MD‐135 sample, while the lowest was in the GA‐135 sample. 2‐Butanol and benzyl alcohol were detected in all powder samples, whereas 2‐ethyl‐1‐hexanol and 2‐phenylethanol were identified only in the GA‐L sample. 2‐Hexanol was found in all samples containing gum arabic, but was absent in those encapsulated solely with maltodextrin (MD‐135 and MD‐L). 1‐dodecanol was detected in all freeze‐dried samples but was not found in spray‐dried samples. 2,3‐butanediol was detected only in the samples dried using spray drying. Notably, 2‐butanol was present at low levels in spray‐dried samples but at higher levels in freeze‐dried ones. These findings suggest that the high temperatures involved in spray drying have a negative effect on volatile alcohol compounds.

**TABLE 4:** Volatile compounds identified in encapsulated cherry laurel powders (peak area × 10^−6^).

	Cherry laurel powder samples						RI
	GA‐135	GAMD‐135	MD‐135	GA‐L	GAMD‐L	MD‐L	
Alcohols							
2‐butanol	0.17 ± 0.02	0.39 ± 0.05	0.30 ± 0.06	6.90 ± 1.05	9.42 ± 0.78	12.65 ± 0.5	603
1‐butanol	ND	0.57 ± 0.04	ND	ND	ND	ND	617
2‐hexanol	1.80 ± 0.35	0.40 ± 0.07	ND	1.26 ± 0.09	0.36 ± 0.04	ND	630
2,3‐butanediol	3.17 ± 0.35	0.95 ± 0.21	2.57 ± 0.98	ND	ND	ND	788
2‐phenylethanol	ND	ND	ND	0.22 ± 0.03	ND	ND	977
2‐ethyl‐1‐hexanol	ND	ND	ND	0.31 ± 0.06	ND	ND	1030
Benzyl alcohol	1.38 ± 0.15	0.34 ± 0.09	13.53 ± 1.7	2.62 ± 0.54	1.82 ± 0.09	2.30 ± 0.13	1040
1‐dodecanol	ND	ND	ND	0.25 ± 0.05	0.20 ± 0.04	0.37 ± 0.08	1323
Total	6.52	6.65	16.4	11.56	11.80	15.32	
Hydrocarbons							
Heptane	ND	3.76 ± 0.17	0.98 ± 0.09	ND	ND	ND	700
Methylbenzene	ND	0.58 ± 0.07	0.39 ± 0.12	ND	ND	ND	763
Dodecane	0.62 ± 0.21	0.45 ± 0.15	1.73 ± 0.17	0.88 ± 0.06	2.18 ± 0.59	1.69 ± 0.21	1200
Nonadecane	ND	ND	0.28 ± 0.07	1.18 ± 0.36	0.33 ± 0.05	0.56 ± 0.23	1292
Tridecane	0.21 ± 0.03	0.18 ± 0.04	0.19 ± 0.03	0.23 ± 0.06	0.51 ± 0.07	ND	1300
Tetradecane	0.68 ± 0.07	0.22 ± 0.01	2.19 ± 0.21	2.88 ± 0.26	3.08 ± 0.78	5.87 ± 1.19	1400
Octadecane	ND	ND	0.20 ± 0.05	0.80 ± 0.23	0.53 ± 0.17	1.67 ± 0.27	1477
Heneicosane	ND	ND	ND	0.55 ± 0.09	0.42 ± 0.02	3.11 ± 0.74	1493
Pentadecane	0.87 ± 0.05	ND	0.13 ± 0.04	ND	ND	ND	1500
Hexadecane	0.49 ± 0.07	0.21 ± 0.04	0.49 ± 0.15	0.94 ± 0.14	2.32 ± 0.97	1.53 ± 0.35	1600
Octacosane	0.88 ± 0.09	ND	ND	ND	ND	ND	2170
Triacosane	1.10 ± 0.21	ND	ND	1.98 ± 0.25	ND	ND	2300
Total	4.85	5.4	6.58	9.44	9.37	14.43	
Ketones							
Acetone	ND	ND	2.18 ± 0.12	ND	ND	ND	628
Acetoin	1.42 ± 0.23	1.93 ± 0.31	2.96 ± 0.21	ND	ND	ND	716
Total	1.42	1.93	5.14	ND	ND	ND	
Esters							
Octyl butanoate	ND	ND	ND	0.48 ± 0.11	ND	1.52 ± 0.17	1389
Methyl benzoate	0.22 ± 0.05	0.07 ± 0.03	0.18 ± 0.04	ND	ND	ND	1589
Total	0.22	0.07	0.18	0.48	ND	1.52	
Acids							
Acetic acid	47.68 ± 2.54	13.53 ± 1.23	7.28 ± 0.59	56.81 ± 3.78	39.69 ± 2.14	2.36 ± 0.23	1043
Total	47.68	13.53	7.28	56.81	39.69	2.36	
Aldehydes							
Acetaldehyde	0.98 ± 0.21	1.11 ± 0.14	1.43 ± 0.23	0.28 ± 0.05	0.63 ± 0.07	0.77 ± 0.08	619
Hexanal	ND	0.30 ± 0.01	0.14 ± 0.04	ND	ND	ND	801
Furfural	ND	ND	ND	0.32 ± 0.02	ND	ND	822
Benzaldehyde	0.33 ± 0.06	2.11 ± 0.11	22.73 ± 2.19	131.37 ± 7.2	111.52 ± 5.65	107.23 ± 6.75	960
Octanal	ND	ND	0.10 ± 0.03	ND	ND	ND	1006
Dodecanal	ND	ND	ND	ND	ND	0.36 ± 0.03	1089
Nonanal	0.31 ± 0.04	0.27 ± 0.04	0.70 ± 0.05	0.98 ± 0.11	0.89 ± 0.09	0.46 ± 0.04	1107
Decanal	ND	ND	ND	0.45 ± 0.03	ND	ND	1208
Total	1.62	3.79	25.1	133.4	113.04	108.82	

*Note:* Values are expressed as mean ± standard deviation.

Abbreviations: ND, not detected; RI, retention index.

Eight volatile aldehydes were identified: acetaldehyde, benzaldehyde, nonanal, furfural, decanal, hexanal, octanal, and dodecanal. The highest total aldehyde content was observed in the GA‐L sample, while the lowest was in the GA‐135 sample. Acetaldehyde, benzaldehyde, and nonanal were detected in all samples; furfural and decanal were exclusive to GA‐L; hexanal was found in GAMD‐135 and MD‐135; octanal in MD‐135 only; and dodecanal in MD‐L only. Benzaldehyde was detected in markedly higher quantities in freeze‐dried samples than in spray‐dried samples, indicating that freeze‐drying was more effective in preserving this aroma‐active aldehyde under the conditions applied. Moreover, among spray‐dried samples, increasing the maltodextrin content correlated with higher benzaldehyde levels, indicating that maltodextrin may reduce thermal degradation. Previous studies have shown that benzaldehyde and hexanal contribute significantly to cherry aroma (Mattheis et al. 1997), and aldehydes are among the most important aroma compounds in sweet cherries (Zhang et al. 2007). Benzaldehyde, derived from enzymatic hydrolysis of amygdalin, is considered the key compound in characteristic cherry aroma. Legua et al. (2017) reported benzaldehyde and nonanal as the most abundant volatiles in Spanish sweet cherry cultivars. Similarly, Hayaloglu and Demir (2016) identified hexanal, 2‐hexenal, nonanal, and benzaldehyde as dominant aldehydes in cherry samples, and Nunes et al. (2022) found benzaldehyde to be the predominant volatile across multiple cherry varieties, contributing to their green and grassy notes.

Dodecane, tetradecane, and hexadecane were the dominant volatile hydrocarbons and were detected in all samples. Overall, freeze‐dried powders exhibited higher hydrocarbon content compared to those produced via spray drying. The highest total hydrocarbon level was found in the maltodextrin‐encapsulated, freeze‐dried sample. Tricosane was detected only in samples encapsulated with 100% gum arabic, while heneicosane was found exclusively in freeze‐dried samples. Ketone compounds were detected only in spray‐dried powders and were absent in freeze‐dried samples, suggesting that the high temperatures involved in spray drying may promote ketone formation. Acetoin was found in all spray‐dried samples, while acetone was detected only in the MD‐135 sample. The highest total ketone content was also observed in the MD‐135 sample, which may be attributed to the effect of maltodextrin as the encapsulating agent. The ester compounds identified in cherry laurel powders are presented in Table 4. Methyl benzoate was detected only in spray‐dried samples, while octyl butanoate was found exclusively in freeze‐dried samples. Among all samples, MD‐L had the highest total ester content. Only acetic acid was identified among the volatile acids. The highest amount of acetic acid was observed in the GA‐L sample, while the lowest was in MD‐L. Acetic acid was found in all powder samples, with higher concentrations in those containing gum arabic. A decrease in gum arabic ratio corresponded to a reduction in acetic acid levels. Mattheis et al. (1997) reported that, in cherries stored for 4 weeks post‐harvest, acetic acid and aldehydes accounted for the majority of detected volatile compounds. In a study on strawberry aroma encapsulation, Balcı‐Torun (2019) found that gum arabic was more effective than maltodextrin or modified starch in encapsulating lipophilic aromatic compounds.

**FIGURE 4:**
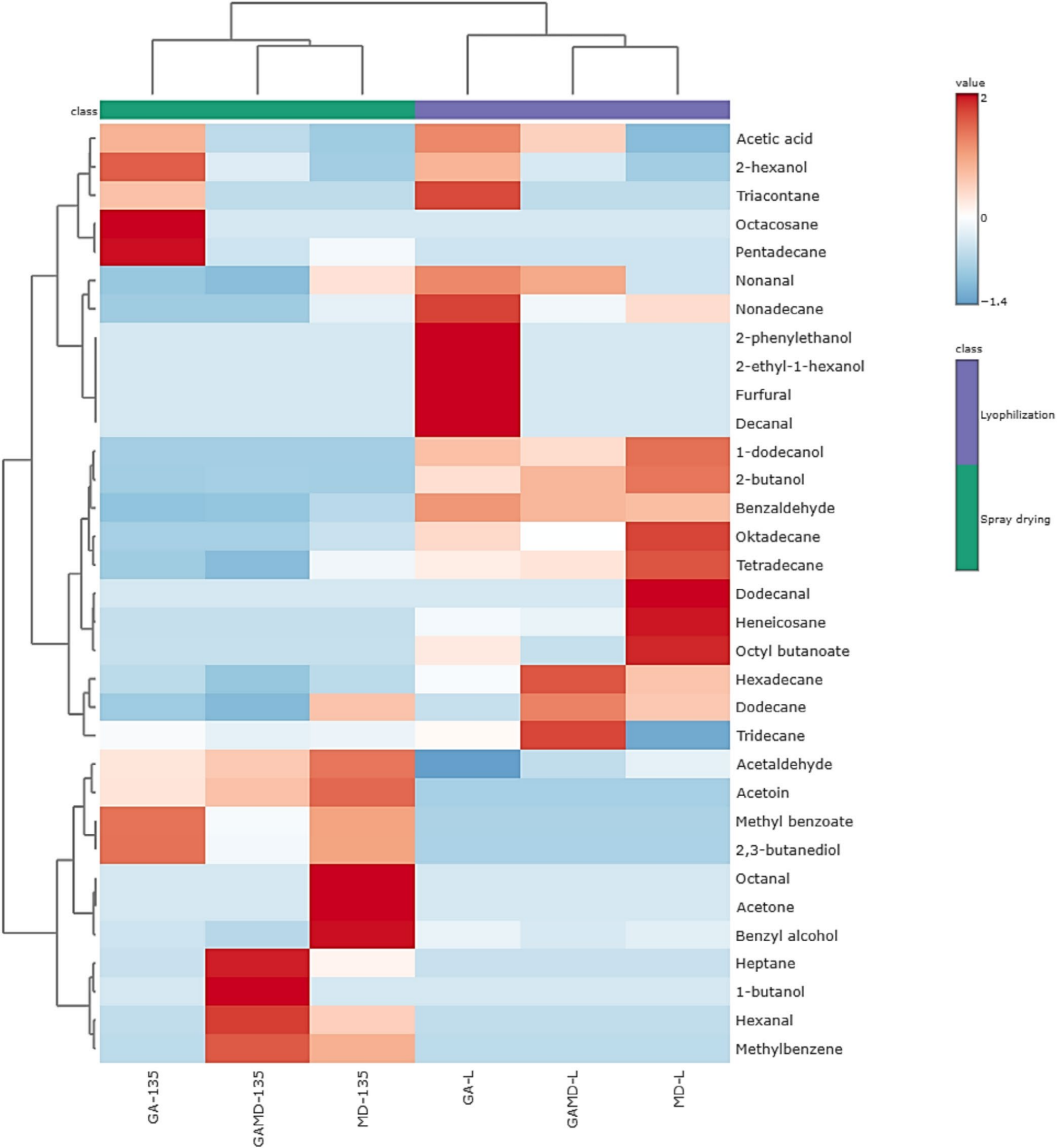
Heatmap of volatile compounds.

**FIGURE 5:**
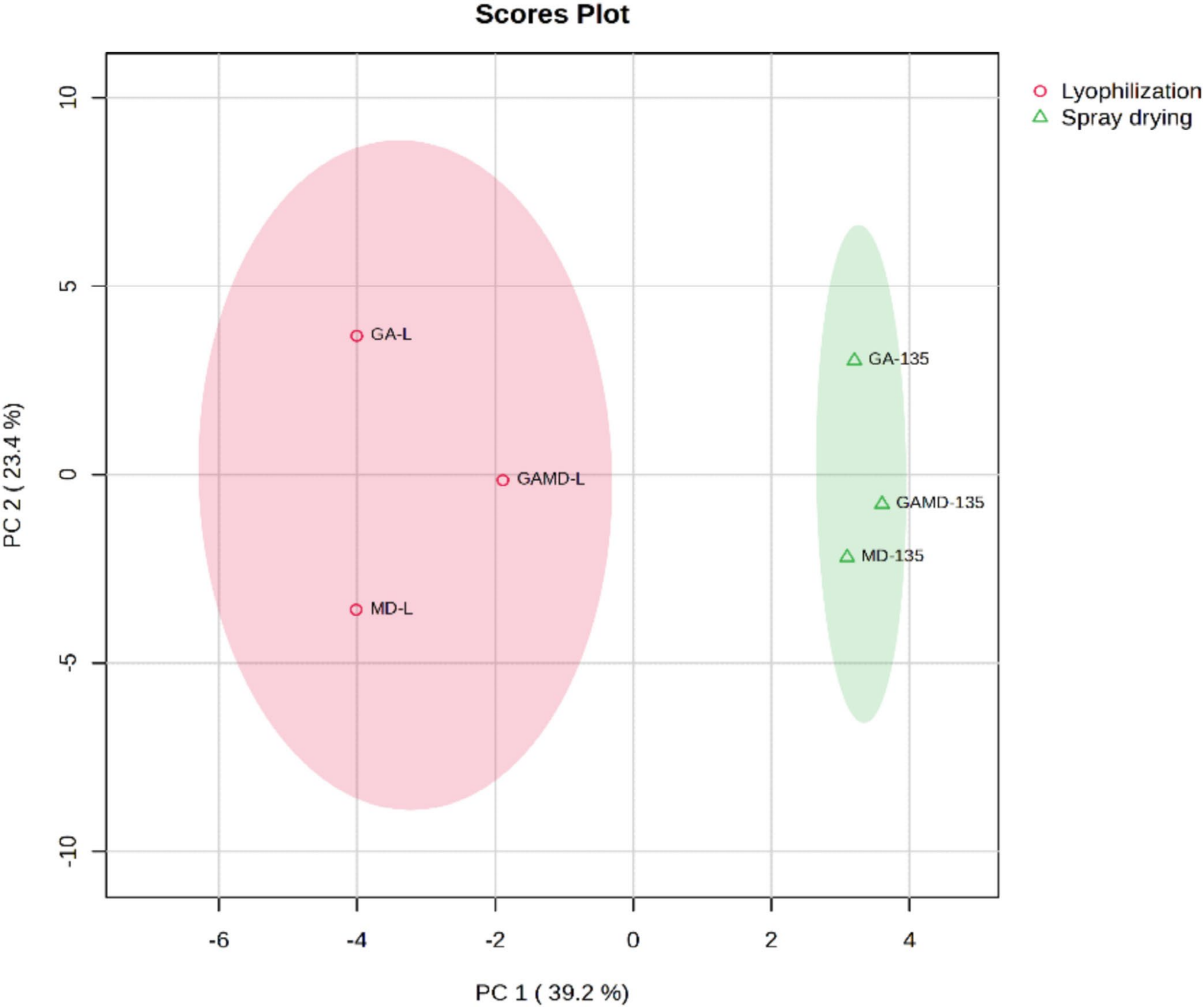
Principal component analysis (PCA) of volatile compounds.

The heatmap and PCA images of the volatile compounds are shown in Figures 4 and 5. The results of the heatmap and PCA analysis collectively highlight the significant impact of the drying technique and encapsulation matrix on the volatile compound profiles of the samples. The heatmap clearly illustrates compound‐specific clustering, where certain volatiles such as 2‐butanol, acetaldehyde, and benzyl alcohol were predominantly found in spray‐dried samples, while compounds such as dodecanal, octyl butanoate, and nonanal were more associated with lyophilized samples. This compound distribution pattern suggests that lyophilization and spray drying distinctly influence the retention and formation of specific aroma compounds.

The PCA score plot further confirms this separation. Samples clustered into two distinct groups based on the drying method, with lyophilized samples (GA‐L, GAMD‐L, MD‐L) located on the left‐hand side of the PCA space and spray‐dried samples (GA‐135, GAMD‐135, and MD‐135) grouped on the right. The first principal component (PC1), which accounts for 39.2% of the variance, appears to be driven largely by the drying technique. The second principal component (PC2) accounts for an additional 23.4%, suggesting a secondary influence—possibly linked to the encapsulation material.

Together, these findings suggest that drying methods do not merely influence the quantity of retained volatiles, but also selectively affect which volatiles are preserved or lost. Furthermore, the close proximity of samples within the same drying category in both analyses indicates that encapsulation materials (GA, MD, and GAMD) may have a more subtle effect compared to the drying method itself.

## 4 Conclusions

Cherry laurel ( *Prunus laurocerasus* ) fruit powders were successfully produced by spray drying and freeze‐drying using gum arabic, maltodextrin, and their mixture as wall materials. Overall, both drying technique and wall material composition influenced powder functionality, bioactive retention, and volatile profiles. The main findings and practical implications are summarized as follows:

- Freeze‐drying resulted in higher powder recovery and higher antioxidant capacity under the conditions tested, suggesting improved preservation of bioactive functionality.
- Spray drying produced powders with more controlled surface morphology, particularly when maltodextrin was used, indicating potential advantages in structural/handling properties depending on the intended application.
- Gum arabic was associated with higher encapsulation efficiency and better retention of phenolic compounds, supporting its suitability when bioactive retention is prioritized.
- Maltodextrin contributed to increased powder lightness (higher L*), consistent with its inherent white color and its effect on visual quality.
- The gum arabic + maltodextrin mixture tended to improve flow properties, with a more pronounced improvement observed in freeze‐dried powders.
- Volatile profiles differed between drying methods: alcohols and hydrocarbons were more prominent in freeze‐dried samples, whereas ketones were mainly associated with spray‐dried powders under the applied conditions, suggesting a possible temperature‐related influence.
- Wall materials also modulated volatile retention: gum arabic tended to favor retention of acetic acid and aromatic esters, while maltodextrin appeared more effective in preserving certain aldehydes and ketones.
- Overall, freeze‐drying may be preferable when the goal is maximizing antioxidant activity and aroma preservation, whereas spray drying—especially with maltodextrin—may offer advantages in physical/structural properties. These findings can guide the development of functional food products enriched with cherry laurel powders tailored to targeted quality attributes (bioactivity, aroma profile, appearance, and handling properties).

## Author Contributions

**Ahmet Alp Karamanoglu:** data curation, investigation, formal analysis, writing – original draft, writing – review and editing, conceptualization. **Zekai Tarakçi:** supervision, methodology, investigation. **Mehmet Akif Karagol:** validation, visualization, writing – review and editing, writing – original draft, investigation, formal analysis.

## Funding

The authors have nothing to report.

## Ethics Statement

This study does not involve any human or animal testing.

## Conflicts of Interest

The authors declare no conflicts of interest.

## Data Availability

The data supporting the results of this study are presented within the article. Further details or Supporting Information can be obtained from the corresponding author upon reasonable request.
